# Long-Term Follow-Up of a Revascularized Immature Necrotic Tooth Evaluated by CBCT

**DOI:** 10.1155/2016/4982458

**Published:** 2016-02-01

**Authors:** C. M. L. She, G. S. P. Cheung, C. F. Zhang

**Affiliations:** Comprehensive Dental Care, Endodontics, Faculty of Dentistry, The University of Hong Kong, Hong Kong

## Abstract

This case study reports the successful treatment of an immature upper premolar with periapical pathosis and sinus tract using revascularization technique. Clinical and radiographic examination demonstrated the recovery of vitality, continued root development, and periapical healing at the 7-month follow-up. In addition, severe calcification of the canal was noted at the 36-month follow-up. At the 66-month follow-up, cone-beam computed tomography (CBCT) revealed complete periapical healing, apical closure, increase in root length and thickness of dentin, and severe calcification of the root canal. Even though the nature of tissue within the root canal is unknown, revascularization appears to give good clinical and radiographic success. This case report highlights that severe calcification of the canal is one of the long-term outcomes of revascularized root canals.

## 1. Introduction

It is often challenging to treat cases of pulpal necrosis involving immature teeth with open apices, as the root canal walls are often thin and prone to fracture. This makes it difficult for root canal instrumentation and obturation [1]. Historically, immature teeth with open apices have been treated by apexification, with placement of intracanal calcium hydroxide medicament to induce the formation of a calcific barrier at the root apex [2]. However, the strong alkalinity of calcium hydroxide may result in the denaturing of the carboxylate and phosphate groups, initiating a collapse of the bonding between the collagen network and hydroxyapatite crystals of the root dentin, thereby increasing the risk of root fracture [3]. Nowadays, apexification is no longer practiced routinely [4].

To overcome the shortcomings of apexification, mineral trioxide aggregate (MTA) has been advocated as the apical root filling to create a barrier [4]. The advantages include shorter treatment time and favorable healing of the periapical tissues due to the good sealing ability and biocompatibility of MTA [2, 5]. However, these teeth are still prone to fracture due to the short root length and insufficient dentin thickness [3, 6].

More recently, revascularization/regenerative procedures have been favored as they allow for the continued growth of immature root. The concept of revascularization is such that it focuses on gaining of blood supply into the disinfected root canal space, triggering a process similar to wound healing in surgical procedures [7]. The primary goal of regenerative endodontic procedures would be to achieve resolution of periapical lesion with absence of any clinical signs or symptoms. The secondary goal would be to achieve an increase in root length and thickness of dentinal walls, along with eventual apical closure. Finally, the tertiary goal would be to obtain positive responses to pulp sensibility testing [8]. There has been a scarcity of long-term case reports in the literature on revascularization of immature teeth with induction of blood clot formation. Most of the published studies have rather short follow-up periods, with the longest reported as 5 years for those involving induction of blood clot formation and 13 years for those without induction of blood clot [9–11]. It is essential to have long-term evaluation of such cases to find out the treatment outcomes, which may include canal calcification.

## 2. Case Report

A 12-year-old male patient with noncontributory medical history was referred to Prince Phillip Dental Hospital, a dental teaching hospital in Hong Kong, for root canal treatment of tooth 15. The patient complained of swelling on the upper right region for the past 3 months. Clinical examination revealed a fractured tubercle (dens evaginatus) of tooth 15. A draining sinus tract was observed on the buccal attached gingiva between tooth 14 and tooth 15. Tooth 15 was tender to percussion and palpation and had negative responses to cold and electric pulp testing. Radiographic examination revealed immature root formation of tooth 15, with a periapical radiolucency measuring approximately 5 mm in diameter. A diagnosis of an open apex with necrotic pulp and chronic apical abscess was made based on the clinical and radiographic findings.

## 3. Treatment

After informed consent was taken, local anesthesia was given using 2% Xylestesin with 1 : 80 000 epinephrine (3M ESPE, Neuss, Germany), and tooth 15 was isolated with a rubber dam. An access cavity was prepared using a high-speed handpiece with copious water spray. The necrotic pulpal remnants were removed under copious irrigation with 3% sodium hypochlorite (15 mL) and sterile saline, along with gentle debridement of the canal walls. Working length was determined by estimating the radiographic working length. The canal was dressed with calcium hydroxide medicament (Calasept, Nordiska Dental, Ängelholm, Sweden) by injecting it directly into the canal and a temporary filling consisting of Cavit (3M ESPE AG, Seefeld, Germany) and Intermediate Restorative Material (IRM) (Dentsply Caulk, Milford, USA) was placed.

At the second visit 3 weeks later, the buccal sinus tract was noted to have healed and the tooth was asymptomatic. The tooth was reopened to replace the calcium hydroxide dressing. At the third visit another 2 weeks later, local anesthesia was given using 3% Mepivastesin (3M ESPE, Neuss, Germany). The root canal was irrigated copiously with sterile saline and dried with sterile paper points (Dentsply Maillefer, Ballaigues, Switzerland). Bleeding was induced by overinstrumentation of the canal length with a size #40 K-file, and a blood clot was formed at the middle third of the root. Grey MTA (ProRoot, Dentsply Tulsa Dental, Johnson City, TN, USA) was placed over the blood clot, followed by a moist cotton pellet above. The access cavity was then restored with IRM (Dentsply Caulk, Milford, USA). The patient was seen 1 week later and the temporary restoration was removed under rubber dam isolation. After confirming that the MTA has set, the tooth was restored with glass-ionomer cement (GC Corporation, Tokyo, Japan) and universal composite resin (3M ESPE, St. Paul, MN, USA).

## 4. Follow-Up Examinations

**Figure 1:**
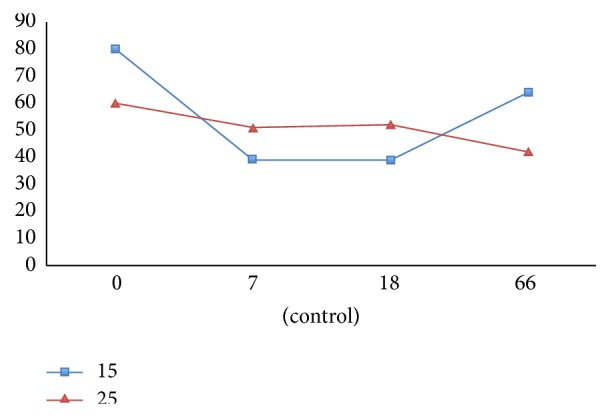
Graph showing the electric pulp testing (EPT) readings of tooth 15 and tooth 25 (control).

**Figure 2:**
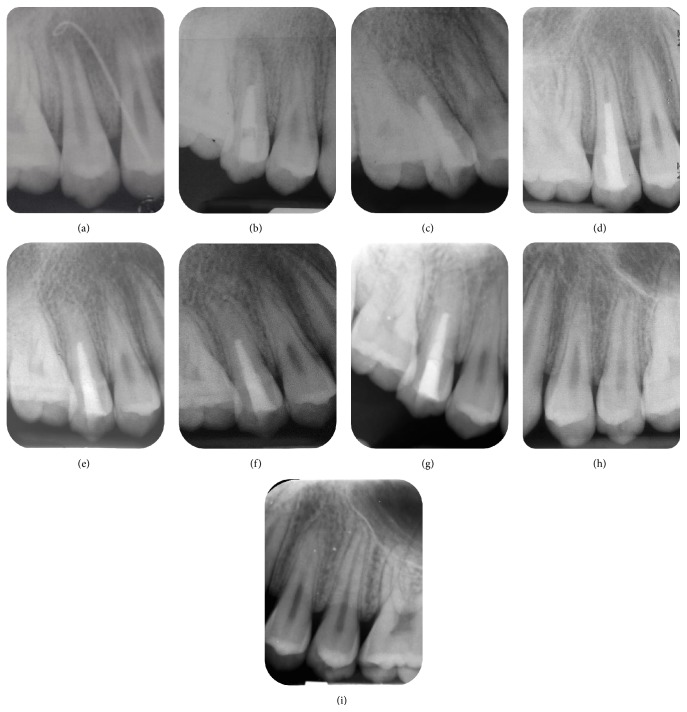
Periapical radiographs. (a) Preoperative, sinus tracing with gutta-percha. (b) After placement of MTA into the root canal. (c) 4-month follow-up showing healing of periapical lesion. (d) 7-month follow-up showing increase in root length. (e) 18-month follow-up showing apical closure. (f) 36-month follow-up showing a narrowed canal lumen. (g) 66-month follow-up showing absence of canal lumen. (h) Tooth 25 (control) at 7-month follow-up. (i) Tooth 25 (control) at 66-month follow-up.

**Figure 3:**
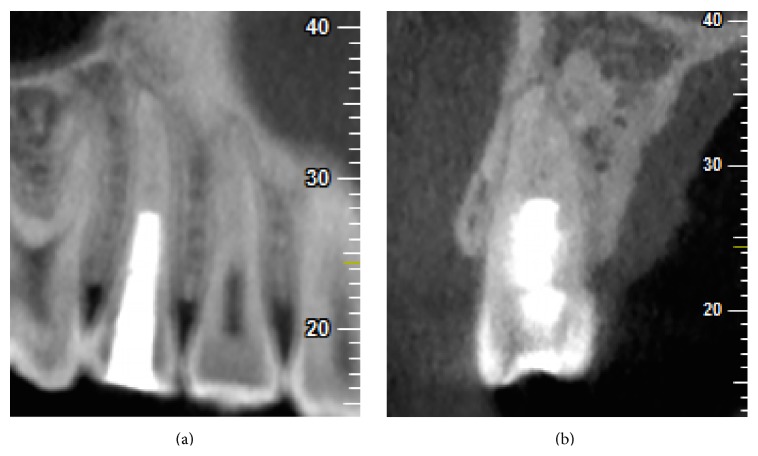
CBCT images at 66 months showing the presence of a narrowed canal lumen due to severe calcifications. (a) CBCT (sagittal view). (b) CBCT (coronal view).

**Figure 4:**
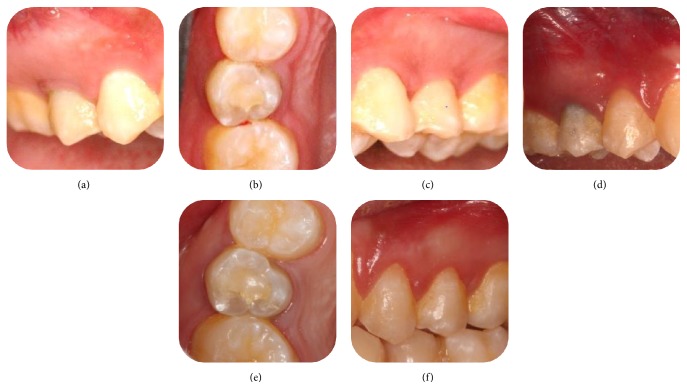
Intraoral photos (1st row: 7-month follow-up, 2nd row: 66-month follow-up). (a) Tooth 15, buccal view. (b) Tooth 15, occlusal view. (c) Tooth 25 (control), buccal view. (d) Tooth 15, buccal view, showing greyish discoloration on the cervical crown of tooth. (e) Tooth 15, occlusal view. (f) Tooth 25, buccal view.

In follow-up visits (at 4, 7, 18, 36, and 66 months postoperatively), no clinical signs or symptoms were detected. Healing of the periapical lesion was observed at the 4-month follow-up. After 7 months, continued root growth was noted, and the tooth gave positive responses to both cold and electric pulp tests (Figure 1). Apical closure was observed at the 18-month follow-up. At the 36-month and 66-month follow-up, periapical radiograph revealed severe calcification of the canal (Figure 2). A CBCT examination of the tooth was done at the 66-month follow-up, which demonstrated a narrowed canal lumen (Figure 3). Clinical examination revealed greyish discoloration at the cervical region of the crown (Figure 4).

## 5. Discussion

Regenerative endodontics has been described as procedures to replace the original structure and histology of damaged or lost dentin, root structures, and cells of the pulp-dentin complex [12]. However, animal histological studies demonstrated the ingrowth of periodontal-like, cementum-like, and bone-like tissues into the pulpal space, rather than true pulpal regeneration [12]. Furthermore, continued root growth after revascularization of immature permanent necrotic teeth depends on the ability of Hertwig's epithelial root sheath (HERS) to survive after apical periodontitis or abscess [13].

Published case reports have shown that successful treatment outcome can be obtained when there is (i) eradication of bacteria from the root canal system, (ii) construction of a scaffold for the ingrowth of new tissue, and (iii) prevention of reinfection by creating a bacteria-tight seal [14]. The choice of irrigants and medicaments has to be made based on their antimicrobial effectiveness, with the minimum destruction to stem cells and growth factors. Both calcium hydroxide and triple antibiotic paste are effective in disinfection of canals [15]. Triple antibiotic paste was used by Banchs and Trope [16], and it has proven to be a very effective antimicrobial agent both in vitro and in vivo [17]. However, it has the disadvantage of causing discoloration of teeth and may be cytotoxic to stem cells at high concentrations. An in vitro study has shown that antibiotic paste with concentrations higher than 1 mg/mL would affect the survival of stem cells in the apical papilla, whereas calcium hydroxide promoted stem cell survival [15]. Hence, recently there seems to be a trend of reverting back to the use of calcium hydroxide intracanal medicament, instead of the triple antibiotic paste.

A limited field-of-view CBCT was used to assess the treatment outcome of this tooth 15 at the 66-month follow-up. It is effective in assessing radiographic changes that may occur in the medullary bone, with the advantage of viewing at various (sagittal, coronal, and transverse) directions [18]. The advantages of CBCT can be clearly seen by comparing the periapical radiograph of tooth 15 with its corresponding CBCT image (Figures 2 and 3), where the former displayed absence of a canal space, while the latter was able to pick up a narrowed canal lumen.

In this case report, severe calcification of canal space was noted, similar to findings reported by other authors [13]. It is one of the five types of treatment outcomes associated with regenerative endodontic procedure reported by Chen et al. [13]. Pulp canal obliteration is a known consequence of traumatic injuries to teeth, tooth replantation, and periodontal disease. However, it is not known if the mechanism is similar in the revascularization of immature permanent teeth with pulp necrosis. The possibility of internal replacement resorption or unification of intracanal solid tissue to apical alveolar bone cannot be ruled out [13]. It is also unknown if there would be any complications associated with pulp canal obliteration, such as pulpal necrosis with apical periodontitis, that has been shown in some teeth affected by traumatic injury [19].

In conclusion, this case report showed good clinical and radiographic outcomes for a revascularization procedure involving an immature tooth with pulpal necrosis and chronic apical abscess. However, clinical success may not equate to histological success. Long-term root canal calcification may be one of the treatment outcomes of revascularized teeth.
